# Interesting Tuberculin Reactions

**Published:** 1897-12

**Authors:** F. S. Schoenleber

**Affiliations:** Assistant State Veterinarian, M’killip Veterinary College, Chicago, Ill.


### INTERESTING TUBERCULIN REACTIONS.

By F. S. Schoenleber, M.S.A., D.V.S.,
ASSISTANT STATE VETERINARIAN, M'KILLIP VETERINARY COLLEGE, CHICAGO, ILL.

During the month of September, 1897, the attention of the State Board of Live-stock Commissioners was called to a herd of dairy-cattle at Hebron, Ill. From the fact that the owner had "lost half a dozen cows during the summer," and from the condition of the remaining portion of the herd, it was evident that tuberculosis was present, although it could be detected in but, perhaps, two or three cases. A tuberculin test was therefore instituted October 26–28.

There were thirty-five milch-cows in the herd, all grade Holsteins and short-horns. Thirty-two were kept in two separate barns. No. 1 contained seventeen head; No. 2 fifteen. The sanitary conditions were, however, quite different in the two cases: barn No. 1 was so filthy that every time an animal moved the slush would ooze out from between the boards of the floor, while barn No. 2 was well kept and comparatively quite clean. Of No. 1, fifteen out of seventeen reacted, or 88.2 per cent.; while of No. 2, only ten out of fifteen, or $66\frac{2}{3}$ per cent., gave evidence of the disease. Whether the percentage of the animals affected in the incipiency of the disease in the two barns in this case had any connection with or relation to the sanitary conditions, of course, cannot be positively stated; however, it looks very suspicious, as there was more or less irregularity in the temperature of nearly all the animals in barn No. 1. The reactions in No. 2 were nothing out of the ordinary. In No. 1 the most conspicuous were the following three:

No. 482, short-horn, weight 1050 pounds; temperature before injection, Oct. 27: 10.30 A.M., 102.1°; 2.30 P.M., 102.2°; 5 P.M., 102.2°. Injected at 7.30 P.M., Oct. 28th; 8 A.M., 106°; 10 A.M., 105.4°; 2 P.M., 98.1°; 4 P.M., 100.2°—maximum increase of 3.3°.

No. 487, short-horn, weight, 1100 pounds; temperature, Oct. 27th, 10.30 A.M., 101.4°; 2.30 P.M., 100.4°; 5 P.M., 101.2°; Oct. 28th, 8 A.M., 104.3°; 10 A.M., 104°; 2 P.M., 102.2°; 4 P.M., 102°—maximum increase of 2.4°.

No. 492, short-horn, weight 1150 pounds; temperature, Oct. 27th, 10.30 A.M., 102°; 2.30 P.M., 101°; 5 P.M., 101.2°; Oct. 28th, 8 A.M., 103.2°; 10 A.M., 104.3°; 2 P.M., 102.4°; 4 P.M., 102°—maximum increase of 2.3°.

The animals were all treated alike; injected at 7.30 P.M., Oct. 27th, and watered at 12 M., Oct. 28th. In No. 482 the temperature at 5 P.M., before injection, was 102.2°; the following morning at 8 o'clock it was 106°, and at 2 P.M., six hours later, it had fallen to 98.1°, or nearly 8 degrees, an average of over one degree per hour for six hours. The deviations from the regular reaction in Nos. 487 and 492 were not quite so great. The condemned cattle (the whole number which reacted) were shipped to the abattoirs at the Chicago stock-yards November 2d and slaughtered under the direction of the State Veterinarian, Dr. C. P. Lovejoy, and the State Board of Live-stock Commissioners, and through their courtesy the autopsies were held by the senior class of the McKillip Veterinary College under the immediate supervision of the dean. The results showed the disease in all in various stages of development, from the localized miliary tubercle in the lung-tissue, or lymphatic gland, to generalized tuberculosis in the viscera and glands, Nos. 482, 487 and 492 presenting no post-mortem deviations from the average of the herd; merely localized tubercles in the lungs and lymphatic glands.

The cows were all in fair flesh, considering the scarcity of pasture during the summer. Gestation had advanced from one to about eight months in most of the herd.

The peculiar deviations in the above temperatures led to a very careful ante- as well as post-mortem inspection; but no further lesions were detected in either. It is regretted that circumstances were such as to prohibit the taking of the temperature in No. 482

for some days following the test. In the face of all the difficulties, however, the results indicate to a great degree how far the test may be relied upon in doubtful or irregular cases, and a study of the thermal lines in the chart made from the temperatures of the animals of the different barns certainly shows a debilitating influence at work somewhere in the system of animals from barn No. 1, due, no doubt, to the filthy condition of the surroundings.

